# Village Life and Industries: A London Show

**Published:** 1919-02-15

**Authors:** 


### VILLAGE LIFE AND INDUSTRIES: a London Show.

By A SOCIAL WORKER.

ARTIST and moralist, architect and social worker, lover of simple life and doctor—all sorts and conditions of English people are taking a new interest in the English village. On all sides it is being eagerly said that city life under modern conditions has become unwholesome and unpleasant, that men from the Army want to continue their outdoor life, that Land Girls want to remain on the land, that cottages require few or no servants—in fact, that the vogue of the Village will be to-morrow's fashion. Possibly in some of those who join in this cry the idea is rather that the village in winter would be a good place for other people to live in. Muddy roads, very long indoor evenings, with darkness outside—would not the mist of dulness and depression creep up and creep in? Is it not the lack of interest, the mental stagnation—plus, of course, the scarcity of cottages—that has in past years driven the flower of village youths and girls to the towns? In old days, no doubt, it was different, and here the mind wanders off to expatiate on the differences.

No one who went to see the exhibition at Caxton Hall of the work done in the 700 village women's Institutes—the Canadian name for an association of women does not imply a building—would readily associate dulness with their life. Gardens, of course, have been common objects of the village. But to realise that the garden can have its value and its interest doubled and trebled by skilled instruction and such devices as co-operative buying of the best seeds, that is to glorify the garden so that drudgery of work there vanishes. The basket of mixed vegetables from Broomfield, in Essex, which won first prize, was commended by the judges for the perfect shape of its straight carrots, significant to the expert, the perfection of shape and skins of potato and Jerusalem artichoke. It needs to be understood, perhaps, that the Board of Agriculture links up and helps the formed institutes as important helpers of national food production. Some excellent specimens of dried fruits were shown. West Malling, Kent, sent a most attractive box of different kinds, and from all parts of the country came cheeses, bottled fruit, and pickles, and specimens of cookery.

The toy industry will not be likely to remain in German hands if the institutes persevere. The soft toys especially showed wonderful variety. "Bath toys" of towelling stuffed with cork and sponge took so many and such attractive forms as geese, ducks, babies, and many other varieties that we are only tempted to wonder whether nurses would easily get their charges to come out again. Soft white dolls and animals to go to bed with were many, and some of the realistic animals extremely clever. A mother kangaroo with pouch and infant kangaroo complete, from Worcestershire, could hardly be bettered. The Sussex "Cuthbert" rabbits have already become well known, and some beautiful ducks made in velvet of their plumage colours came from Dorsetshire. Some highly artistic work was shown in lace of many kinds. The opportunity for a revival of old crafts should be seized before these are forgotten. A most interesting exhibit was a rug done in artistic shading by a charwoman at Swaffham. This was the proper old method of rag-carpet making, each strand brought to the front of the knitting and looped over a thick pin, producing a number of neat and *un*ragged "knobs" of the same size. A really beautiful hand-made carpet of thrums copied the design of a Roman pavement in the worker's own neighbourhood.

Weaving appeared also, and raises an interesting question: Will the Board of Education restore spinning to its proper position and let it be taught in schools? Wool from the hedges had been spun by one exhibitor with a *distaff* and crocheted into a bonnet. But it needs some eight spinners to supply a wool or linen loom with thread. And the possibilities of fillet lace work for discharged soldiers are under inquiry. Thread for them would be needed, and Ireland is again growing much flax.

Shoes are soled by women in many places. Rabbit-skins are shown beautifully cured. Herbs are dried, and some admirable home-dyeing was shown, most artistic shades being produced in fawns, greys, pinks, etc., by the use of lichen, privet, and sloe. Hats were prettily made of plaited paper, and the uses of waste shown in many ways. When gloves threaten to reach prohibitive prices it is cheering to see what a pretty and cosy fingerless pair can be produced from the top of an old sock for the palm, the ribbed part shaping well to the wrist, while the back is covered with a well-dressed rabbit-skin.

The Queen honoured the show with a visit, and if the promoters have anything to regret it can only be that more space was not available both for the exhibits on the stalls and for the circulation of the crowds.

